# Innate immune responses to malaria-infected erythrocytes in pregnant women: Effects of gravidity, malaria infection, and geographic location

**DOI:** 10.1371/journal.pone.0236375

**Published:** 2020-07-29

**Authors:** Marzieh Jabbarzare, Madi Njie, Anthony Jaworowski, Alexandra J. Umbers, Maria Ome-Kaius, Wina Hasang, Louise M. Randall, Bill Kalionis, Stephen J. Rogerson

**Affiliations:** 1 Department of Medicine at the Doherty Institute, University of Melbourne, Melbourne, Australia; 2 School of Health and Biomedical Sciences, RMIT University, Melbourne, Victoria, Australia; 3 Vector Borne Diseases Unit, Papua New Guinea Institute of Medical Research, Madang, Papua New Guinea; 4 Department of Maternal-Fetal Medicine, Pregnancy Research Centre, Royal Women’s Hospital Department of Obstetrics and Gynaecology, University of Melbourne, Parkville, Australia; Instituto Rene Rachou, BRAZIL

## Abstract

**Background:**

Malaria in pregnancy causes maternal, fetal and neonatal morbidity and mortality, and maternal innate immune responses are implicated in pathogenesis of these complications. The effects of malaria exposure and obstetric and demographic factors on the early maternal immune response are poorly understood.

**Methods:**

Peripheral blood mononuclear cell responses to *Plasmodium falciparum*-infected erythrocytes and phytohemagglutinin were compared between pregnant women from Papua New Guinea (malaria-exposed) with and without current malaria infection and from Australia (unexposed). Elicited levels of inflammatory cytokines at 48 h and 24 h (interferon γ, IFN-γ only) and the cellular sources of IFN-γ were analysed.

**Results:**

Among Papua New Guinean women, microscopic malaria at enrolment did not alter peripheral blood mononuclear cell responses. Compared to samples from Australia, cells from Papua New Guinean women secreted more inflammatory cytokines tumor necrosis factor-α, interleukin 1β, interleukin 6 and IFN-γ; p<0.001 for all assays, and more natural killer cells produced IFN-γ in response to infected erythrocytes and phytohemagglutinin. In both populations, cytokine responses were not affected by gravidity, except that in the Papua New Guinean cohort multigravid women had higher IFN-γ secretion at 24 h (p = 0.029) and an increased proportion of IFN-γ^+^ Vδ2 γδ T cells (p = 0.003). Cytokine levels elicited by a pregnancy malaria-specific CSA binding parasite line, CS2, were broadly similar to those elicited by CD36-binding line P6A1.

**Conclusions:**

Geographic location and, to some extent, gravidity influence maternal innate immunity to malaria.

## Introduction

Malaria in pregnancy is responsible for 10,000 maternal deaths annually [1] and is associated with increased risk of miscarriage, stillbirth, fetal growth restriction, preterm deliveries, low birth weight (LBW) and infant mortality [2–5]. These complications are particularly associated with placental malaria, characterized by the attachment of *Plasmodium falciparum-*infected erythrocytes (IE) to chondroitin sulfate A (CSA) expressed on the placental syncytiotrophoblast. This placental adhesion is mediated by a member of the *P*. *falciparum* erythrocyte membrane protein-1 (PfEMP1) family called *VAR2CSA*. The severity of maternal and fetal complications varies and is influenced by factors including gravidity, malaria endemicity and exposure [6, 7].

Partial immunity against clinical malaria develops with repeated parasite exposure. In low and unstable malaria transmission regions, all pregnant women are at risk and *P*. *falciparum* infections can rapidly lead to severe maternal, fetal and infant complications [7]. In contrast adults, including pregnant women, living in high and stable transmission areas acquire a substantial level of immunity and generally experience asymptomatic infections or mild malaria symptoms [4]. They generally have previous exposure to a wide variety of PfEMP1 variants, which mediate adhesion to endothelial receptors such as CD36, intercellular adhesion molecule 1 and endothelial protein C receptor.

In malaria-endemic areas, susceptibility to *P*. *falciparum* infections is relatively higher in first pregnancy (primigravidae) than in later pregnancies (multigravidae) [8]. The average prevalence of antenatal infections in Papua New Guinea (PNG) was approximately two-fold higher in primigravidae compared to multigravidae [9, 10]. Since primigravidae have little or no prior exposure to *VAR2CSA* expressing parasites, they lack sufficient *VAR2CSA* neutralizing antibodies and are consequently more prone to PM-associated complications [9, 10].

While the effects of malaria transmission intensity and gravidity are mainly associated with antibody-mediated immunity, their potential impact on maternal innate immunity is not well understood. As essential components of innate immune responses, inflammatory cytokines, natural killer (NK) cells and γδ T cells are of particular interest in maternal and neonatal immunity. NK cells can directly lyse target cells, activate antigen-presenting cells and promote Th1 responses [11] while the exposure of γδ T cells to IE in vitro results in early Th1 cytokine responses [12, 13]. However, the roles of both NK and γδ T cells in either protection from, or the pathogenesis of, *P*. *falciparum* malaria are not clear [12, 14–16].

In normal pregnancy Th1 cytokine responses are generally suppressed, but PM can result in placental elevation of tumor necrosis factor α (TNF-α), which is associated with both anemia [17] and LBW [17, 18], while increased interferon γ (IFN-γ) levels could mediate either protective [19] or pathogenic [17] effects. Increased levels of the chemokines interleukin 8 (IL-8), monocyte chemoattractant protein 1 (MCP-1), macrophage inflammatory protein 1 (MIP-1) α and MIP-1 β are crucial in parasite clearance but potentially harmful to pregnancy as they can enhance placental infiltration of immune cells such as monocytes [20, 21], which have been associated with anemia and LBW [22]. With these positive and negative associations, the magnitude and response profile of inflammatory mediators could be important determinants of pregnancy outcomes in malaria in pregnancy.

In a previous study from PNG, cytokine and chemokine secretion by peripheral blood mononuclear cells (PBMC) stimulated with VAR2CSA-expressing CS2-IE were reported to differ between multigravidae (2–4 pregnancies) and grand-multigravidae (5–7 pregnancies) [23]. However, it remains unclear whether the observed immune response was affected by lifetime malaria exposure or gravidity. The present study, therefore, investigates the effect of gravidity, lifetime malaria exposure, active malaria infection and different *P*. *falciparum* strains on maternal innate immune responses.

## Materials and methods

### Ethics approval and consent to participate

Ethical approvals were obtained from the Royal Women’s Hospital Human Research Ethics Committee (project 08/33), the Medical Research Advisory Council of Papua New Guinea, the Melbourne Health Human Research Ethics Committee and Alfred Health Human Research Ethics Committee.

All participants provided written informed consent prior to enrolment.

### Study locations, participants and sample collection

Primigravid and multigravid women were recruited from health facilities in Madang Province PNG, and the Royal Women's Hospital, Melbourne, Australia (AUS). PNG is a malaria endemic region, with a high intensity of rainfall and malaria transmission as previously described [24]. Recruitment in PNG occurred between September 2005 and October 2007 [25]; Exclusion criteria included hemoglobin <5 g/dL; permanent disability and chronic medical conditions; known multiple pregnancy; unavailable for follow-up; and age <16 years.

**Fig 1:**
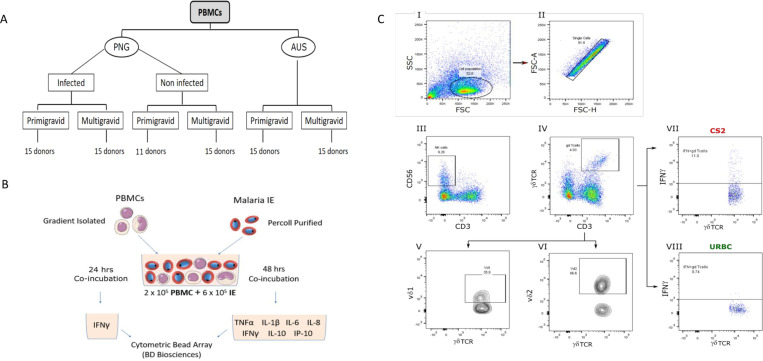
(A) Stratification of study population: PBMC derived from pregnant women from Papua New Guinea (PNG) and Australia (AUS) were categorized into six test groups based on sample origin, gravidity and current malaria infection. From PNG: infected primigravidae (15), infected multigravidae (15), non-infected primigravidae (11) and non-infected multigravidae (15). From Australian: primigravidae (15) and multigravidae (15). (B) Schematic representation of PBMC stimulation assay: PBMC were isolated by Ficoll gradient centrifugation and frozen at -80°C. PBMC were thawed in batches and co-incubated with Percoll-purified infected erythrocytes (IE), phytohemagglutinin (PHA) or uninfected red blood cells (uRBC) for 24 or 48 hours. At 24 hrs, supernatants were measured for IFNγ, and cells harvested, stained and analyzed for intracellular staining. At 48 hrs, supernatant were analyzed for TNF-α, IFN-γ, IL-1β, IL-6, IL-8, IL-10, and IP-10 by cytometric bead array. (C) Gating strategy for NK cells, γδ T cells and two distinct populations of γδ T cells (Vδ1^+^ and Vδ2^+^) producing IFN-γ. Live lymphocytes were identified using forward scatter (FSC) and side scatter (SSC) (I) before excluding cell doublets (II). Then, NK cells (CD3^-^CD56^+^) (III), γδ T cells (CD3^+^ TCR^+^) (IV), Vδ2 γδ T cells (γδTCR^+^ Vδ2^+^) (V) and Vδ1 γδ T cells (γδTCR^+^ Vδ1^+^) (VI) were gated on. Finally, the proportion (%) of each cell type producing IFN-γ was determined (e.g. proportion of IFNγ producing γδ T cells in response to CS2 IE (VII) and uRBC (VIII). Quadrants defining positive and negative cell populations were set according to single color controls. FACS plots are from one representative donor.

At enrolment (14–26 gestational weeks) and prior to treatment, 10 ml peripheral blood was collected in sodium heparin to isolate PBMC. Malaria infection was determined by peripheral blood microscopy and a polymerase chain reaction/ligase detection reaction-fluorescent microsphere assay (LDR-FMA) [25] which detected *P*. *falciparum*, *P*. *vivax* and *P*. *ovale*. Based on these data, study samples were categorized into malaria positive and negative by light microscopy and then grouped by gravidity. The Australian cohort comprised malaria-naïve pregnant women who had been scheduled for elective caesarean section. Exclusion criteria were hypertension, gestational or pre-existing diabetes, and obesity (body mass index >30 kg/m^2^). At enrolment (>38 gestation weeks), 10 ml peripheral blood was collected in ethylenediaminetetraacetic acid (EDTA) tubes for PBMC isolation within two hours of collection. PBMC samples were categorized into six test groups based on exposure, gravidity and current malaria infection as shown in Fig 1A.

### Parasite culture and preparations

The laboratory-adapted *P*. *falciparum* lines CS2 (which binds to CSA and mimics pregnancy associated isolates) [26] and P6A1 (a clone of the A4 parasite line that binds to CD36, gift of Professor Joe Smith [27]) were cultivated at 5% hematocrit with human blood group O/Rhesus positive red blood cells (RBC) from healthy volunteers provided by the Australian Red Cross blood service (Agreement number 16-02VIC-06) as described in [28]. Cultures were screened regularly to exclude Mycoplasma contamination using MycoAlert™ mycoplasma detection kit (Lonza) [29]. To ensure that the respective binding phenotype of the parasite lines were maintained, cultures were tested for adhesion to immobilized receptors and panned on immobilised CSA (CS2) or CD36 (P6A1) as described in [30]. Cultures were synchronised by gelatine flotation for the selection of knobby trophozoite stage parasites every 1 to 2 weeks [31]. Prior to stimulation assays, mature trophozoite IE were purified by density gradient centrifugation using layers of 80%, 60% and 40% Percoll in supplemented RPMI 1640-HEPES as described elsewhere [32, 33].

### PBMC isolation

At both sites, following whole blood dilution with warm RPMI 1640 (Gibco), PBMC were isolated by density gradient centrifugation (700xg, 25 min) using Ficoll-Paque (GE Healthcare). Cells were harvested and if RBC contamination was observed, samples were incubated in 0.2% NaCl (37°C, 3 min) to lyse RBC. PBMC were then washed in cold Ca^2+^/Mg^2+^ free phosphate-buffered saline (PBS) and aliquots frozen in 20% DMSO in fetal bovine serum (FBS) at -80°C [34].

### PBMC elicitation assay

PBMC samples were stimulated in duplicates with CS2 or P6A1 IE, uninfected RBCs (uRBC), and mitogenic lectin phytohemagglutinin (20 μg/mL PHA, Life Technologies) as illustrated in Fig 1B. Briefly, PBMC were thawed at 37°C and washed twice (700xg, 10 min, room temperature) with PBMC medium (RPMI 1640, 10% FBS, 25 mM HEPES, 2 mM L-glutamine, 100 U/mL penicillin, 100 μg/mL streptomycin). Cell viability was assessed by trypan blue staining. In 96-well round bottom plates, 2 x 10^5^ viable cells/well were plated and rested at 37°C for 1 hour. PBMC were then incubated with IE (6 x 10^5^ per well), uRBC (6 x 10^5^ per well) or PHA (2%), and supernatants were analysed after 48 hours [35, 36]. PBMCs were tested in batches of 6–9 samples at a time, selected from across the different participant groups.

### Cytokine measurement

A multiplex assay (Cytometric Bead Array; Flex Sets; BD Biosciences) was used to quantify cytokine levels according to the manufacturer’s instructions. In pre-wetted 96-well round bottom plates, a set of antibody-coated capture beads specific for human TNF-α, IFN-γ, IL-1β, IL-6, IL-8, IL-10, and IP-10 were premixed and incubated with supernatants and standards. Subsequent steps were carried out according to the manufacturer’s instructions. Bead suspensions were analysed using a CyAn flow cytometer (Beckman Coulter) and fluorescence intensity was converted to cytokine concentration using the FCAP array (version 3) or Hypercyte software. The assay sensitivity for each cytokine was: 1.2 pg/mL (TNF-α), 1.8 pg/mL (IFN-γ), 2.3 pg/mL (IL-1β), 1.6 pg/mL (IL-6), 1.2 pg/mL (IL-8), 0.13 pg/mL (IL-10) and 0.5 pg/mL (IP-10) [37].

### Intracellular staining for IFN-γ

PBMCs were stimulated with CS2 IE or uRBC for 24 h at 37°C in 5% CO_2_. (Cell numbers were insufficient to also test P6A1). Brefeldin A (0.1 μg/mL, eBioscience) was added 6 h before harvesting the cells. Cells were washed (200xg, 4 minutes, 4°C) with FACS buffer (2 mM EDTA and 1% Heat Inactivated FBS (HI-FBS) in PBS) and stained with a panel of surface markers [anti-CD3ε PerCP Cy5.5 (1:200; clone SP34-2, BD), CD56 APC (1:25; clone N901, Beckman Coulter), γδ TCR (1:50; clone 11F2, BD), TCR Vδ2 BV711 (1:200; clone B6, BD or BioLegend), TCR Vδ1 FITC (1:50; clone TS8.2, Thermo Scientific)] for 30 min on ice. All antibodies were used at empirically determined dilutions that provided optimum signal-to-noise ratio. Cells were washed twice with FACS buffer and stained with a fixable viability dye (aqua live/dead amine-reactive dye; Invitrogen) for 15 min at RT for dead cell exclusion. Intracellular staining [IFN-γ PE (1:100; clone 4S.B3, BD)] was performed using a BD Cytofix/Cytoperm ICS kit (BD Biosciences) as per the manufacturer's instructions. A minimum of 10000 events were acquired on a 4-laser Fortessa flow cytometer (BD Biosciences, CA) and data were analysed using FlowJo software version 10. Using the combination of fluorescence minus 1 (FMOs) and single colour controls (Ultra Comp eBeads, eBioscience) as a gating strategy, the proportions of IFN-γ^+^ cells (i.e. NK and γδ T cells) were determined [36].

### Statistical analysis

Statistical analysis was performed using GraphPad Prism (Version 5). Data were tested for normality using Shapiro-Wilks and Kolmogorov-Smirnov normality tests. Paired samples were compared using Wilcoxon signed-rank test or Student's t-test as appropriate. Unpaired samples were compared using the Mann-Whitney U test or t-test. A probability value of <0.05 was considered significant. Results are presented as median and interquartile range (IQR), and absolute values rounded up to integers.

## Results

### Clinical characteristics of the study cohort

Thirty women from Australia (AUS), 30 women from PNG with malaria infection (PNG_I) and 26 women from PNG without malaria infection (PNG_N) were included in this study. The lower number in the PNG_N group was due to limited sample number. AUS and PNG_I groups were each divided into 15 primigravid and 15 multigravid women, whiles the PNG_N group constituted 11 primigravid and 15 multigravid women. Table 1 compares the clinical characteristics of the study groups. The median (interquartile range) of maternal hemoglobin [13 (11–14) vs 8 (6–10) g/dL], maternal age [33 (30–48) vs 24 (17–36) years] and gestational age at sample collection [39 (38–40) vs 25 (19–33) weeks] were significantly higher in Australian women compared to malaria-negative PNG women. Of the infected PNG women 17 had parasites detected by microscopy and PCR, while 13 had submicroscopic infection. Twenty infections were with *P*. *falciparum* while ten were with *P*. *vivax* and/or *P*. *ovale*.

**Table 1:** Clinical characteristics and stratification of study groups.

	PNG_I		PNG_ N		AUS		*P value* (PNG_I vs PNG_N) *	*P value* (PNG_N vs AUS) *
	n = 30		n = 26		n = 30			
**Maternal age at enrolment (years)**	22 (16–32)		24 (17–36)		33 (30–48)		0.826	**< 0.001**
**Maternal hemoglobin level (g/dL)**	8.2 (5–11)		8 (6–10)		13 (11–14)		0.928	**< 0.001**
**Recruitment gestational age (weeks)**	25 (7–30)		25 (19–33)		39 (38–40)		0.563	**< 0.001**
	**PNG_I**		**PNG_N**		**AUS**			
**Stratification by gravidity**	**Primi**	**Multi**	**Primi**	**Multi**	**Primi**	**Multi**		
	n = 15	n = 15	n = 11	n = 15	n = 15	n = 15		

Data are median (interquartile range). **PNG**: Papua New Guinea; **I**: Malaria infected women; **N**: Non-malaria infected women; **AUS**: Australia; **Primi:** Primigravidae; **Multi:** Multigravidae

*: Mann-Whitney U Test; ***P values* in Bold**: Statistically significant.

### Effect of anticoagulant on cytokine responses

Because blood was collected in PNG in heparin, and in Melbourne it was collected in EDTA, we compared cytokine elicitation from PBMCs collected from 4 donors which were collected into each of the two anticoagulants. No statistically significant differences were seen in cytokine elicitation between cells collected in the differing anticoagulants (S1 Fig).

### Effect of current malaria infection on cellular activity and cytokine responses

To assess the influence of current malaria infection on the responsiveness of PBMC, PNG women were segregated according to active malaria infection at study enrolment as shown in Table 2. Upon stimulation with CS2 IE, cytokine responses were similar between the two groups except for IFN-γ which was moderately higher in the non-infected group at 48 h (p = 0.026) but not at 24 h. Consistent with the 24 h observation, the proportions of IFN-γ-secreting cells (NK and γδ T cells) were not different between the malaria-infected and non-infected groups.

**Table 2:** Cytokine secretion and cellular responses to CS2 IE between malaria infected and non-infected women from PNG.

	PNG_ I	PNG_ N	P value*
	n = 30	n = 26	
	Cytokine levels at 48 hours (pg/ml)		
**TNF-α**	7 (2–53)	14 (2–32)	0.993
**IFN-γ**	90 (6–385)	329 (134–1006)	**0.026**
**IL-1β**	8 (2–26)	7 (2–20)	0.954
**IL-6**	136 (29–1028)	149 (55–621)	0.902
**IL-8**	10535 (5808–20020)	14407 (9384–20637)	0.475
**IL-10**	14 (6–28)	15 (5–40)	0.844
**IP-10**	298 (120–903)	650 (77–2442)	0.320
**IFN-γ** **^^**	78 (6–567)	166 (52–545)	0.349
	Percentage of IFN-γ+ cells at 24 hours		
**NK cells**	12 (3–23)	7 (4–13)	0.320
**γδ T cells**	11 (4–17)	7 (5–11)	0.366
**Vδ2 T cells**	11 (4–21)	9 (4–18)	0.889
**Vδ1 T cells**	9 (2–17)	4 (2–12)	0.175

Data are median (interquartile range). **PNG**: Papua New Guinea; **I**: Malaria infected women; **N**: Non-malaria infected women

*: Mann-Whitney U Test; ***P value* in Bold**: Statistically significant

**^^**: Cytokine level at 24 hours.

### Effect of malaria exposure on cellular activity and cytokine responses

To evaluate the effect of sample origin (i.e. PNG and Australia) on innate cellular responses, PBMC responses to CS2 and P6A1 IE were compared between Australian participants and malaria-uninfected PNG women as noted in Table 3. Compared to those from Australia, PBMCs from PNG produced significantly higher levels of all tested cytokines in response to both parasite lines, including IFN-γ at both 24 h and 48 h. The proportion of IFN-γ^+^ NK cells was somewhat higher (P = 0.063) in PBMC from PNG compared to samples from Australia, but no significant differences in proportions IFN-γ^+^ cells were seen. Patterns of cytokine secretion following stimulation with P6A1 IE were broadly similar to those using CS2 IE (Table 3), except that there were lower levels of TNF-α (Australia), IL-8 (both PNG and Australia) and IP-10 (PNG) produced following stimulation with P6A1 compared to CS2 (S1 Table).

**Table 3:** Cytokine secretion and cellular responses to malaria-IE in PNG and Australian women.

	CS2-IE			P6A1-IE		
	PNG_N	AUS	*P value* *	PNG_N	AUS	*P value* *
	n = 26	n = 30		n = 26	n = 30	
	Cytokine levels at 48 hours (pg/ml)					
**TNF-α**	14 (2–32)	4 (2–25)	**<0.001**	10 (3–48)	2 (1–6)	**<0.001**
**IFN-γ**	328 (132–1005)	16 (1–72)	**<0.001**	334 (113–1044)	10 (1–44)	**<0.001**
**IL-1β**	7 (2–20)	1 (1–1)	**<0.001**	5 (2–39)	1 (1–1)	**<0.001**
**IL-6**	149 (55–620)	7 (2–17)	**<0.001**	90 (26–1244)	4 (1–18)	**<0.001**
**IL-8**	14407 (9384–20637)	535 (102–1676)	**<0.001**	13167 (4459–16867)	215 (65–1080)	**<0.001**
**IL-10**	14 (5–40)	1 (1–1)	**<0.001**	18 (4–38)	1 (1–1)	**<0.001**
**IP-10**	650 (77–2442)	30 (1–100)	**<0.001**	489 (32–1437)	5 (1–50)	**<0.001**
**IFN-γ** **^^**	166 (52–545)	24 (7–93)	**<0.001**	129 (51–588)	22 (7–47)	**<0.001**
	Percentage of IFN-γ+ cells at 24 hours					
**NK cells**	11 (6–16)	8 (2–14)	0.063			
**γδ T cells**	7 (4–10)	8 (3–11)	0.837			
**Vδ2 T cells**	9 (4–18)	8 (3–13)	0.240			
**Vδ1 T cells**	4 (2–12)	7 (3–15)	0.246			

Data are median (interquartile range). **PNG**: Papua New Guinea; **N**: Non-Malaria Infected women; **AUS**: Australia

*: Mann-Whitney Test; ***P values* in Bold**: Statistically significant

**^^**: Cytokine level at 24 hours.

### Effect of gravidity on cellular activity and cytokine responses

PBMC derived from malaria-negative PNG women and Australian women were categorised by gravidity, and their responses to CS2 IE compared in Table 4. In both study cohorts, cytokine secretion at 48 h was similar between primigravid and multigravid women. However, for the 24-hr time point, a significantly higher IFN-γ secretion was observed in multigravid compared to primigravid women from PNG (347 versus 54 pg/mL; p = 0.029). A similar trend was also observed in the Australian cohort. When intracellular cytokine staining was assessed, the proportion of IFN-γ-producing γδ T cells (p = 0.013) and especially the Vδ2^+^ subset (p = 0.003) were substantially increased in multigravid women compared to primigravid women from PNG. In contrast, the proportions of IFN-γ-producing cells were similar between primigravid and multigravid women from Australia. Patterns of cytokine secretion following stimulation with P6A1 IE did not differ between primigravid and multigravid women (S2 Table).

**Table 4:** Cytokine secretion and cellular responses to CS2 IE between primigravid and multigravid women.

	PNG_N			AUS		
	Primi	Multi	*P value* *	Primi	Multi	*P value* *
	n = 11	n = 15		n = 15	n = 15	
	Cytokine levels at 48 hours (pg/ml)					
**TNF-α**	20 (3–24)	8 (2–94)	0.621	1 (1–3)	1 (1–4)	0.999
**IFN-γ**	208 (145–607)	541 (8–1092)	0.716	10 (1–60)	19 (1–77)	0.900
**IL-1β**	7 (2–8)	11 (3–67)	0.391	1 (1–1)	1 (1–1)	0.351
**IL-6**	89 (66–204)	291 (34–804)	0.755	4 (2–24)	8 (3–17)	0.561
**IL-8**	13805 (10746–18785)	16906 (1990–24330)	0.640	293 (72–1131)	893 (112–1805)	0.384
**IL-10**	12 (6–31)	18 (2–53)	0.211	1 (1–1)	1 (1–1)	0.609
**IP-10**	698 (282–1613)	478 (16–3078)	0.406	1 (1–117)	33 (1–94)	0.387
**IFN-γ** **^^**	54 (7–385)	347 (124–615)	**0.029**	16 (3–38)	45 (10–105)	0.097
	IFN-γ levels and Percentage of IFN-γ+ cells at 24 hours					
**NK cells**	10 (6–12)	14 (7–18)	0.213	8 (2–13)	8 (1–16)	0.836
**γδ T cells**	5 (3–6)	8 (7–16)	**0.013**	7 (4–10)	9 (2–15)	0.967
**Vδ2 cells**	4 (3–6)	12 (9–24)	**0.003**	7 (3–10)	9 (2–16)	0.520
**Vδ1 cells**	2 (1–10)	8 (2–15)	0.096	10 (4–15)	6 (2–15)	0.534

Data are median (interquartile range). **PNG**: Papua New Guinea; **N**: Non-malaria infected women; **AUS**: Australia

*: Mann-Whitney Test; **Primi:** Primigravidae; **Multi:** Multigravidae; ***P values* in Bold**: Statistically significant

**^^**: Cytokine level at 24 hours.

### Cellular responses to non-malaria stimuli

To determine if the observations were specific to IE, cellular responses to PHA and uRBC were compared between PNG and Australian women as shown in Table 5. In response to PHA, elicited levels of TNF-α, IL-1β, IL-6, IL-8 and IL-10 (48 h: p<0.001 for all) as well as IFN-γ (24 h: p<0.001) were significantly higher in samples from PNG compared to those from Australia. Additionally, the proportion of IFN-γ-producing NK cells (p = 0.024) was significantly higher in PNG women compared to the Australian group. Furthermore, IFN-γ (p = 0.029), IL-6 (p = 0.005) and IL-8 (p = 0.002) responses to uRBC were significantly higher in samples from PNG compared to those from Australia.

**Table 5:** Cytokine secretion and cellular responses to PHA and uRBC.

	PHA			uRBC		
	PNG_N	AUS	*P value* *	PNG_N	AUS	*P value* *
	n = 26	n = 30		n = 26	n = 30	
	Cytokine levels at 48 hours (pg/ml)					
**TNF-α**	754 (19–1323)	1 (1–9)	**<0.001**	1 (1–1)	1 (1–1)	0.942
**IFN-γ**	2 (1–4057)	38 (1–148)	0.953	7 (2–240)	1 (1–1)	**0.006**
**IL-1β**	36(2–97)	1 (1–1)	**<0.001**	1 (1–1)	1 (1–1)	0.061
**IL-6**	1241 (34–5622)	5 (2–13)	**<0.001**	8 (4–88)	1 (1–1)	**<0.001**
**IL-8**	7338 (2932–14059)	732 (134–1474)	**<0.001**	14 (1–83)	1 (1–3)	**0.002**
**IL-10**	69 (1–183)	1 (1–1)	**<0.001**	1 (1–1)	1 (1–1)	0.061
**IP-10**	1 (1–987)	5 (1–93)	0.677	1 (1–1)	1 (1–1)	0.115
**IFN-γ** **^^**	1369 (390–3416)	35 (7–68)	**<0.001**	0.1 (0.1–0.2)	0.1 (0.1–3)	0.874
	Percentage of IFN-γ+ cells at 24 hours					
**NK cells**	8 (6–19)	4 (1–14)	**0.024**	0.1 (0.1–1)	0.1 (0.1–1.0)	0.992
**γδ T cells**	3 (2–17)	5 (2–18)	0.593	0.1 (0.1–2)	0.1 (0.1–1.4)	0.440
**Vδ2 T cells**	3 (2–20)	2 (0.3–15)	0.243	0.1 (0.1–2)	0.1 (0.1–1.4)	0.954
**Vδ1 T cells**	7 (3–19)	9 (1–22)	0.954	0.1 (0.1–3)	0.1 (0.1–1.3)	0.960

Data are median (interquartile range). **PHA**: Phytohemagglutinin; **uRBC**: Uninfected erythrocytes; **PNG**: Papua New Guinea; **N**: Non-malaria infected women; **AUS**: Australia

*: Mann-Whitney Test; ***P values* in Bold**: Statistically significant

**^^**: Cytokine level at 24 hours.

## Discussion

This study used an *in vitr*o approach to explore how active malaria infection, lifetime malaria exposure and gravidity influence early cellular immune responses, comparing pregnant women from PNG and Australia. When IE were used as the stimulus, active malaria infection did not substantially alter responses, but PBMCs from malaria-exposed PNG women secreted more inflammatory cytokines, and more of their NK cells produced IFN-γ^+^ than the malaria-naïve Australian cohort. Patterns of IFN-γ production and secretion with exposure to pregnancy-associated IE showed some gravidity-dependent differences in PNG women, indicating a potential role for acquired immunity. Higher cytokine secretion in PNG women than Australian women in response to PHA stimulation suggested broader immune activation in PNG. Geography and gravidity may affect the responsiveness of maternal innate cells to malaria IE and other stimuli.

Malaria infection, confirmed by both microscopy and qPCR, was not associated with general differences in inflammatory cytokine responses. Although cells from non-infected women produced higher levels of IFN-γ at 48 h than cells from infected women, this difference was not apparent at 24 h. In addition, the proportions of IFN-γ producing NK and γδ T cells were not different between these groups, and secretion of no other tested cytokines differed. These data suggest that the functional activity and inflammatory responses of maternal PBMC are not substantially altered by concurrent exposure to malaria parasites. Although we cannot exclude the possibility of occult placental malaria, in agreement with our results, the proportion of NK cells and IFN-γ production in peripheral blood were reported to be similar between infected and non-infected pregnant women in Western Kenya [38].

On exposure to CS2 IE, PBMC derived from PNG pregnant women had higher proportions of functional NK cells and produced stronger inflammatory responses than samples obtained from Australian residents. The reasons for the marked differences in cytokine secretion between the two groups are not known. It has been proposed that host genetic determinants such as the NK-cell killer immunoglobulin-like receptor repertoire can influence early IFN-γ responses to *P*. *falciparum* IE [16, 39], and differences in genetic background between the two cohorts might explain the difference in cellular responses, but would require further investigation. A further possibility is trained immunity, induced either by exposure to malaria [40] or to other stimuli [41], and resulting in transcriptional or epigenetic reprogramming of innate immune cells to produce more active inflammatory responses. The lower levels of some cytokines seen in both PNG and Australian women using P6A1 IE rather than CS2 IE may reflect the ability of CD36-binding IE to be taken up by non-opsonic phagocytosis through CD36 on monocyte-lineage cells, with potentially inhibitory effects on cytokine secretion [42].

One potential implication of the differences in cytokine secretion may be a greater ability to control parasitemia in the PNG cohort, perhaps indicating a form of functional immunity, indicated by the increased functional responsiveness of NK cells and the rapid release of inflammatory mediators in this group [38]. In line with this proposition, decreased activity of NK cells and consequently insufficient IFN-γ production has been associated with severe placental malaria episodes [43, 44]. On the other hand, this could imply the vulnerability of PNG women to adverse pregnancy outcomes, as elevations in cytokines such as TNF-α in placental malaria have been correlated with LBW [17, 18, 21].

Although the elevated cellular responses observed with the PNG samples could be related to prior malaria exposure, a similar pattern was observed with a non-malaria stimulus, PHA, (and, to some extent with incubation with uRBC) suggesting that heightened responsiveness was not malaria-specific, but may instead indicate greater immune activation in PNG than Australian women. Possible factors underlying this difference might include exposure of PNG women to a diverse range of communicable diseases, such as helminth and bacterial infections [45]. Unfortunately, participants were not tested for infection with these pathogens, which might have an impact on the responsiveness of the innate cellular repertoire to stimuli such as IE and PHA [46]. This proposed general immune activation might overshadow the specific effects of prior malaria exposure.

The increased susceptibility of primigravid women to malaria is partly explained by the gravidity dependent acquisition of protection from placental malaria which is associated with the acquisition of antibodies against *VAR2CSA* which opsonises IE infected with pregnancy-specific parasites [47, 48] and prevents their placental sequestration. Studies examining how cellular responses to IE may differ with gravidity are relatively few in number. In our study, secretion of inflammatory mediators at 48 h was not influenced by gravidity in either study cohort, but among women from PNG, elicited levels of IFN-γ at 24 h were significantly higher in multigravid compared to primigravid women. This is consistent with a previous study from Kenya using placental mononuclear cells [19]. The higher secretion was associated with a higher proportion of IFN-γ producing γδ T cells, specifically the Vδ2^+^ subset, in multigravid women compared to primigravid women. Recent studies suggest that γδ T cells are essential for non-specific targeting of *P*. *falciparum* [49] and can show a “memory/recall” phenotype [50–52]. With increased exposure to CSA-binding IE over successive pregnancies, women are presumed to develop increased pregnancy-malaria specific IFN-γ responses. We did not have sufficient cells to examine effects of CD36-binding IE on intracellular staining for IFN-γ, and so could not confirm whether the gravidity dependent differences observed in PNG women are restricted to pregnancy-associated IE.

In adults, γδ T cells particularly Vδ2^+^ Vγ9 γδ T cells express high levels of CD45RO which is thought to exhibit a memory phenotype [53]. A higher proportion of CD45RO memory-like T cells was reported in PM-negative multigravid women compared to women in earlier pregnancies [38], suggesting that it may be part of a memory immune response that could improve anti-parasite immunity. However, CD45RO expression was not evaluated in the present study and would be interesting for future studies to investigate.

Strengths of the study include testing PBMC from pregnant women from PNG and Australia, matched for gravidity, side by side with identical stimuli. Although PBMCs constitute a significant proportion of adaptive immune cells, the stimulation timepoints (24–48 h) potentially exclude significant contributions of adaptive immune cells to cytokine levels. As other studies have observed [54, 55], the early (24–48 h) response from PBMCs reflects mainly activation of innate immune cells including monocytes, NK cells and γδ T cells, while acquired responses only became significant after 72 h or more in culture. Among PNG women, those without malaria infection were negative by both microscopy and highly sensitive qPCR. No women in either cohort had recognised immune disturbances. Possible study weaknesses include differences in gestation, maternal age and hemoglobin levels at the time of sample collection, and differences in anticoagulant used, although the choice of anticoagulant was shown not to affect cytokine responses to IE. Pregnancy is generally divided into an early implantation-related inflammatory state, a prolonged quiescent state which persists throughout most of pregnancy, and the return to an inflammatory state only at onset of parturition [56]. Of note, none of the Melbourne women were in active labour, and gestation is not known to significantly alter innate immune responses. We did not have sufficient material to test cells for evidence of epigenetic modifications characteristic of trained immunity [41]. Our analysis did not correct for multiple comparisons due to the exploratory nature of the study, and our sample size was relatively small (with 26–30 women in most groups), limiting our power to detect differences between groups, and to analyse any differences in responses according to infecting species. Finally, we did not routinely test participants for human immunodeficiency virus infection, but the prevalence of HIV infection in ANCs in the region was only 1% at the time of sample collection (Unger, HW, Bolnga JW; unpublished data).

## Conclusions

In summary, the present study demonstrates that geographic origin and to some degree gravidity can influence maternal innate immune responses to malaria IE. Women from PNG exhibit rapid and higher inflammatory cytokine responses to both malaria and non-malaria stimuli (IEs and PHA) than an Australian cohort, suggesting a broader immune activation and perhaps an indication of functional immunity for PNG inhabitants. Furthermore, the expansion of functional Vδ2 γδ T cells in multigravidae compared to primigravidae in PNG suggested a potential memory phenotype and a role in gravidity-dependent protection against clinical malaria. Host genetics, immune activation or epigenetic changes characteristic of trained immunity, may explain the substantially higher inflammatory cytokine production, and the modest increase in numbers of IFN-γ producing NK cells in PNG women compared to Autralian pregnant women.

## Supporting information

S1 Fig Effect of anticoagulant (EDTA and Heparin) on PBMC cytokine secretion. (PPTX)

S1 Table Comparison of cytokine responses to CS2 and P6A1. (PPTX)

S2 Table Cytokine responses to P6A1 IE in primigravidae and multigravidae. (PPTX)

## List of abbreviations

AUS: Australia
CSA: Chondroitin sulfate A
EDTA: Ethylenediaminetetraacetic acid
IE: Infected erythrocytes
IFN-γ: Interferon γ
IL-8: Interleukin 8
IPTp: Intermittent preventive treatment in pregnancy
LBW: Low birth weight
MIP-1: Macrophage inflammatory protein 1
MCP-1: Monocyte chemoattractant protein 1
NK: Natural killer
PNG: Papua New Guinea
PBMC: Peripheral blood mononuclear cell
PfEMP1: *P*. *falciparum* erythrocyte membrane protein-1
PHA: Phytohemagglutinin
RBC: Red blood cells
TNF-α: Tumor necrosis factor α
