# Supplementary material for: Innate immune responses to malaria-infected erythrocytes in pregnant women: Effects of gravidity, malaria infection, and geographic location
Source: PLoS One. 2020 Jul 29;15(7):e0236375. doi: 10.1371/journal.pone.0236375 (PMC7390391; doi:10.1371/journal.pone.0236375)
Supplement: S1 Fig — (PPTX) [file pone.0236375.s001.pptx]

## Slide 1
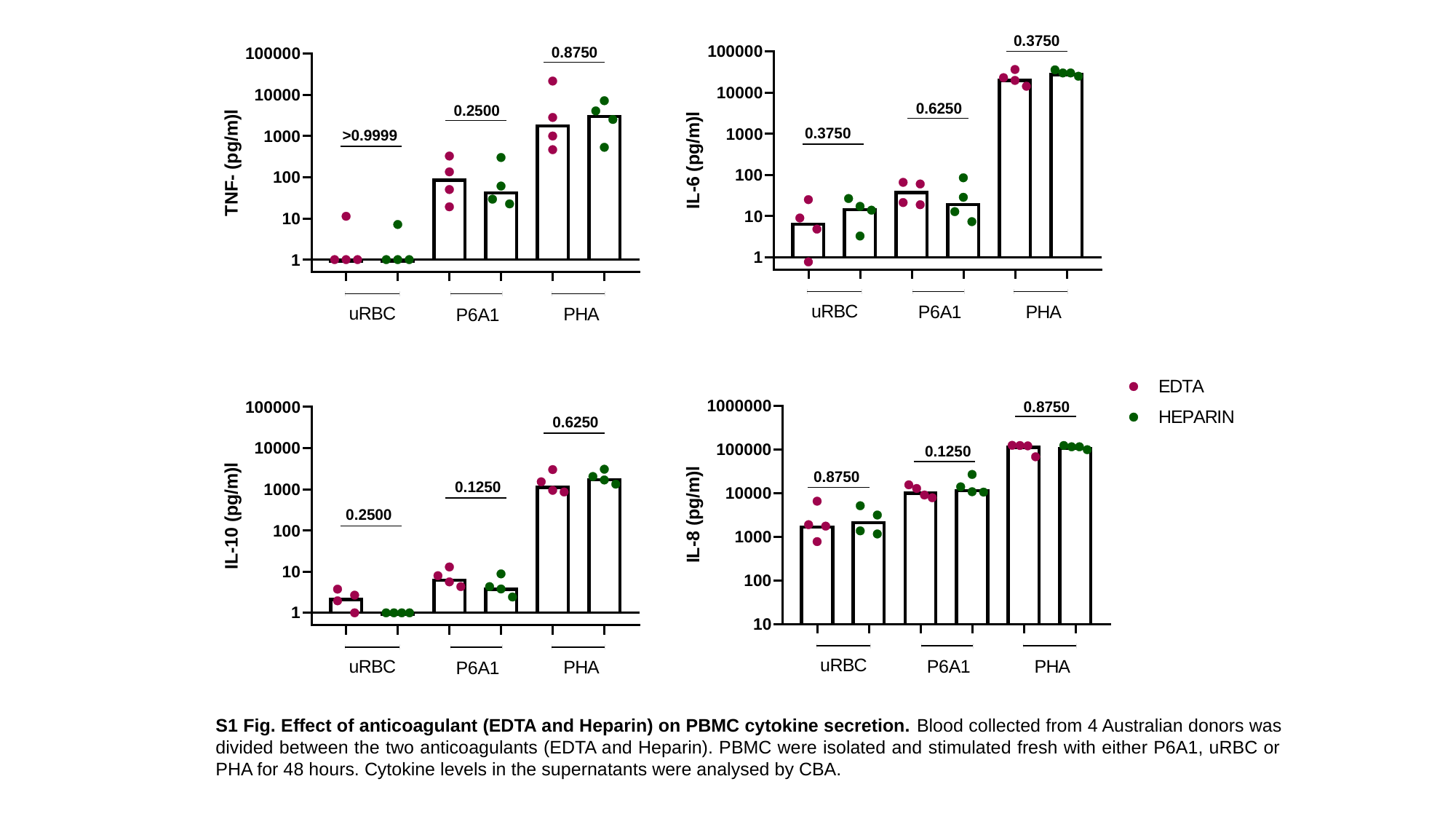

S1 Fig. Effect of anticoagulant (EDTA and Heparin) on PBMC cytokine secretion. Blood collected from 4 Australian donors was divided between the two anticoagulants (EDTA and Heparin). PBMC were isolated and stimulated fresh with either P6A1, uRBC or PHA for 48 hours. Cytokine levels in the supernatants were analysed by CBA.
