# Supplementary material for: Innate immune responses to malaria-infected erythrocytes in pregnant women: Effects of gravidity, malaria infection, and geographic location
Source: PLoS One. 2020 Jul 29;15(7):e0236375. doi: 10.1371/journal.pone.0236375 (PMC7390391; doi:10.1371/journal.pone.0236375)
Supplement: S1 Table — (PPTX) [file pone.0236375.s002.pptx]

## Slide 1
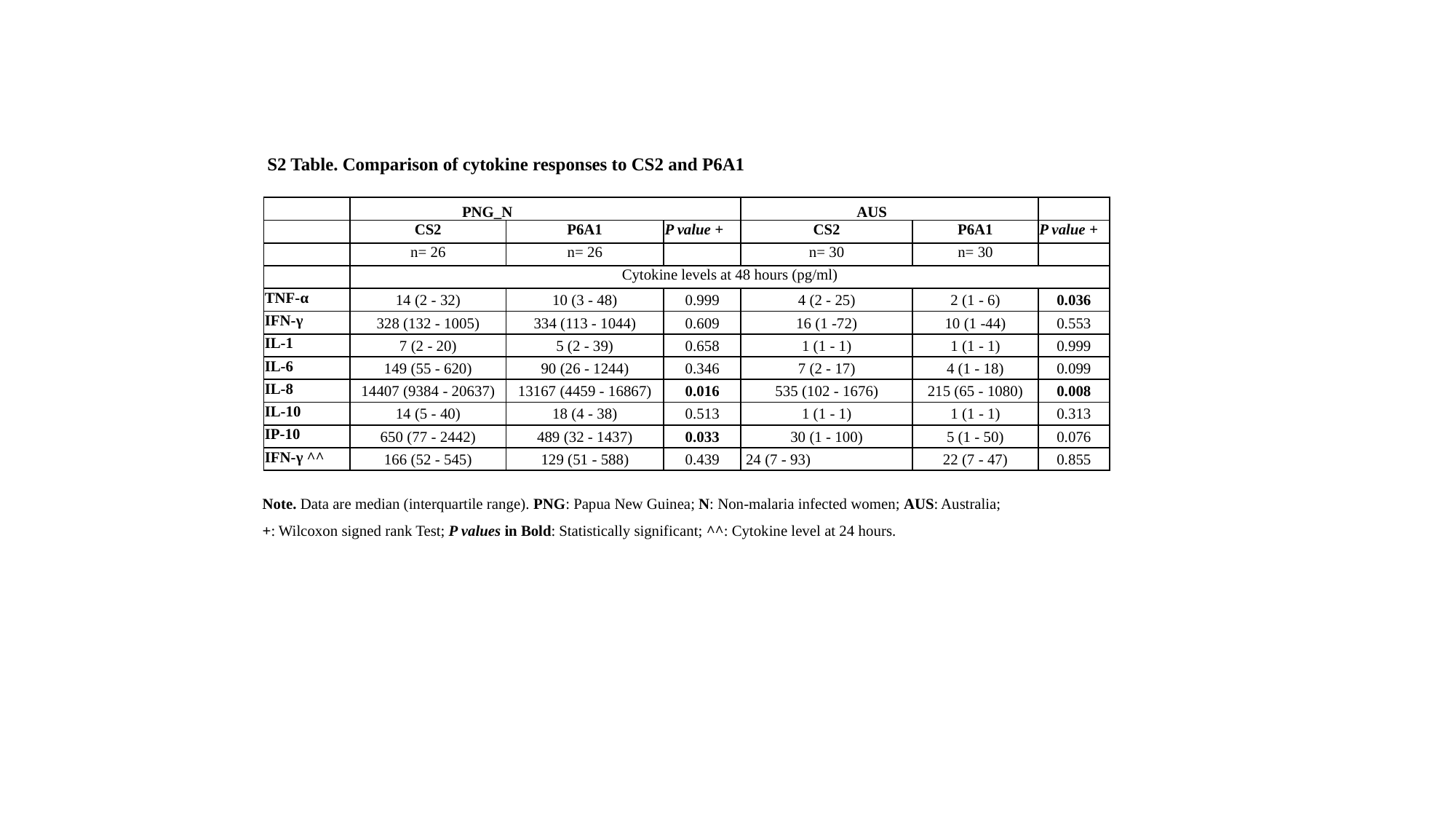

S2 Table. Comparison of cytokine responses to CS2 and P6A1
| | PNG\_N | | | AUS | | |
| --- | --- | --- | --- | --- | --- | --- |
| | CS2 | P6A1 | P value + | CS2 | P6A1 | P value + |
| | n= 26 | n= 26 | | n= 30 | n= 30 | |
| | Cytokine levels at 48 hours (pg/ml) | | | | | |
| TNF-α | 14 (2 - 32) | 10 (3 - 48) | 0.999 | 4 (2 - 25) | 2 (1 - 6) | 0.036 |
| IFN-γ | 328 (132 - 1005) | 334 (113 - 1044) | 0.609 | 16 (1 -72) | 10 (1 -44) | 0.553 |
| IL-1 | 7 (2 - 20) | 5 (2 - 39) | 0.658 | 1 (1 - 1) | 1 (1 - 1) | 0.999 |
| IL-6 | 149 (55 - 620) | 90 (26 - 1244) | 0.346 | 7 (2 - 17) | 4 (1 - 18) | 0.099 |
| IL-8 | 14407 (9384 - 20637) | 13167 (4459 - 16867) | 0.016 | 535 (102 - 1676) | 215 (65 - 1080) | 0.008 |
| IL-10 | 14 (5 - 40) | 18 (4 - 38) | 0.513 | 1 (1 - 1) | 1 (1 - 1) | 0.313 |
| IP-10 | 650 (77 - 2442) | 489 (32 - 1437) | 0.033 | 30 (1 - 100) | 5 (1 - 50) | 0.076 |
| IFN-γ ^^ | 166 (52 - 545) | 129 (51 - 588) | 0.439 | 24 (7 - 93) | 22 (7 - 47) | 0.855 |
Note. Data are median (interquartile range). PNG: Papua New Guinea; N: Non-malaria infected women; AUS: Australia;
+: Wilcoxon signed rank Test; P values in Bold: Statistically significant; ^^: Cytokine level at 24 hours.
