# Supplementary material for: Innate immune responses to malaria-infected erythrocytes in pregnant women: Effects of gravidity, malaria infection, and geographic location
Source: PLoS One. 2020 Jul 29;15(7):e0236375. doi: 10.1371/journal.pone.0236375 (PMC7390391; doi:10.1371/journal.pone.0236375)
Supplement: S2 Table — (PPTX) [file pone.0236375.s003.pptx]

## Slide 1
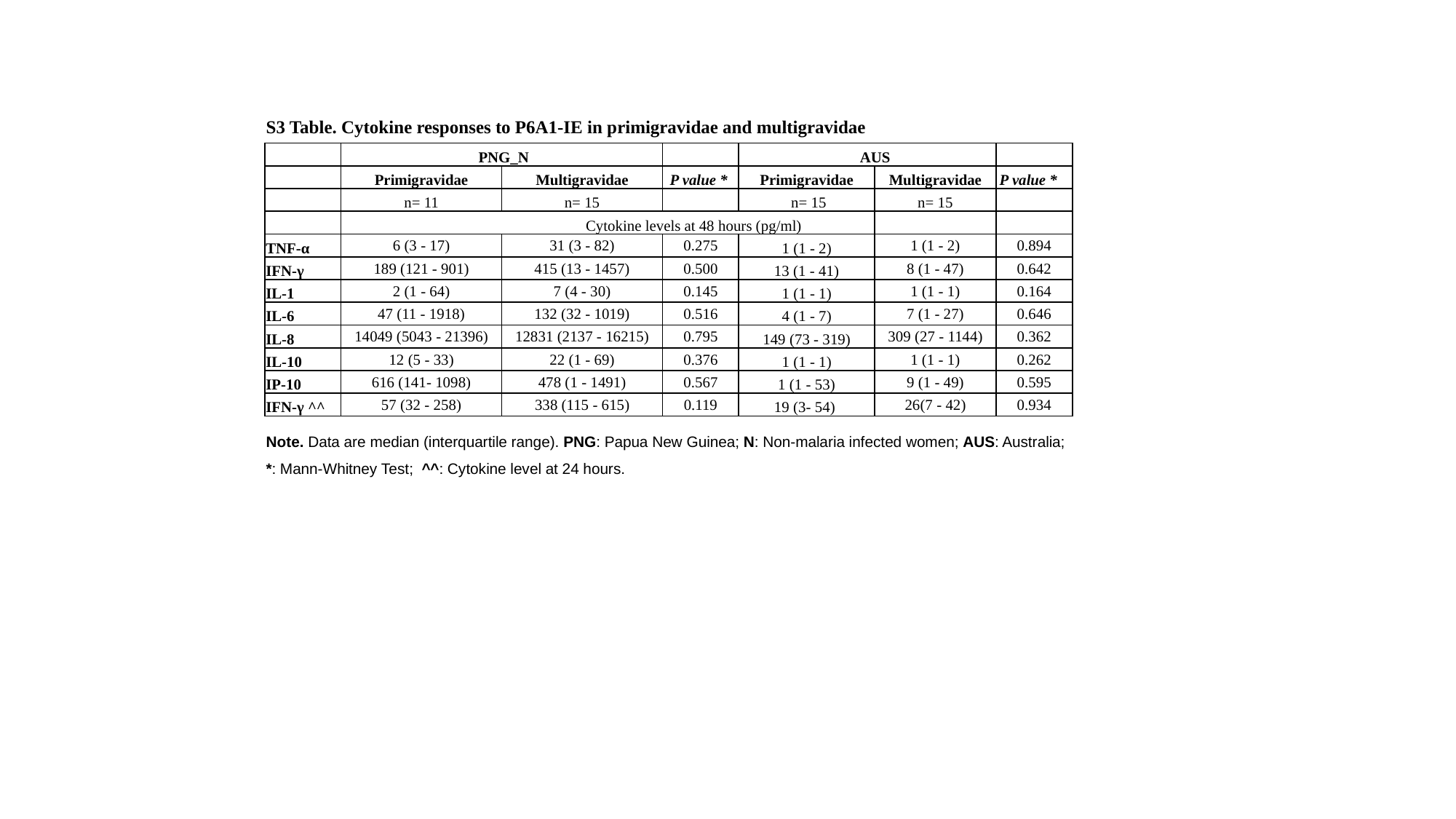

S3 Table. Cytokine responses to P6A1-IE in primigravidae and multigravidae
| | PNG\_N | | | AUS | | |
| --- | --- | --- | --- | --- | --- | --- |
| | Primigravidae | Multigravidae | P value \* | Primigravidae | Multigravidae | P value \* |
| | n= 11 | n= 15 | | n= 15 | n= 15 | |
| | Cytokine levels at 48 hours (pg/ml) | | | | | |
| TNF-α | 6 (3 - 17) | 31 (3 - 82) | 0.275 | 1 (1 - 2) | 1 (1 - 2) | 0.894 |
| IFN-γ | 189 (121 - 901) | 415 (13 - 1457) | 0.500 | 13 (1 - 41) | 8 (1 - 47) | 0.642 |
| IL-1 | 2 (1 - 64) | 7 (4 - 30) | 0.145 | 1 (1 - 1) | 1 (1 - 1) | 0.164 |
| IL-6 | 47 (11 - 1918) | 132 (32 - 1019) | 0.516 | 4 (1 - 7) | 7 (1 - 27) | 0.646 |
| IL-8 | 14049 (5043 - 21396) | 12831 (2137 - 16215) | 0.795 | 149 (73 - 319) | 309 (27 - 1144) | 0.362 |
| IL-10 | 12 (5 - 33) | 22 (1 - 69) | 0.376 | 1 (1 - 1) | 1 (1 - 1) | 0.262 |
| IP-10 | 616 (141- 1098) | 478 (1 - 1491) | 0.567 | 1 (1 - 53) | 9 (1 - 49) | 0.595 |
| IFN-γ ^^ | 57 (32 - 258) | 338 (115 - 615) | 0.119 | 19 (3- 54) | 26(7 - 42) | 0.934 |
Note. Data are median (interquartile range). PNG: Papua New Guinea; N: Non-malaria infected women; AUS: Australia;
*: Mann-Whitney Test; ^^: Cytokine level at 24 hours.
